# Use of π‑Space CASSCF Trial Wave Functions in DMC Calculations of Unsaturated Organic Molecules

**DOI:** 10.1021/acs.jpca.6c02019

**Published:** 2026-07-20

**Authors:** Nastasia Mauger, Kenneth D. Jordan

**Affiliations:** Department of Chemistry, University of Pittsburgh, Pittsburgh, Pennsylvania 15218, United States

## Abstract

The major source of error in fixed-node diffusion Monte Carlo (DMC) electronic structure calculations is the inadequate description of the nodal surface for electron exchange. In this work, we examine the use of π-space complete active space self-consistent field (CASSCF) trial wave functions for several unsaturated hydrocarbons and their derivatives. We find that reoptimizing the CI coefficients of the CASSCF wave function in the VMC step together with parameters in the Jastrow factor leads to substantial lowering of the DMC energies. In general, this reoptimization causes significant reduction in magnitude of the coefficients of the minor configurations in wave functions. Moreover, it is shown for five of the systems studied that one can obtain a similar energy lowering by simply scaling all secondary configurations by a factor of ∼0.5. It is further demonstrated that one can predict, often to within chemical accuracy, the energy lowering when going from single-determinant DMC to CAS-DMC based on the magnitude of the dominant coefficient of the trial wave function, both across different molecules and along structural distortions.

## Graphical abstract

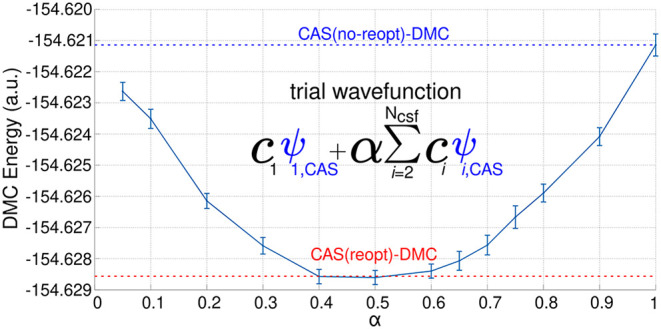

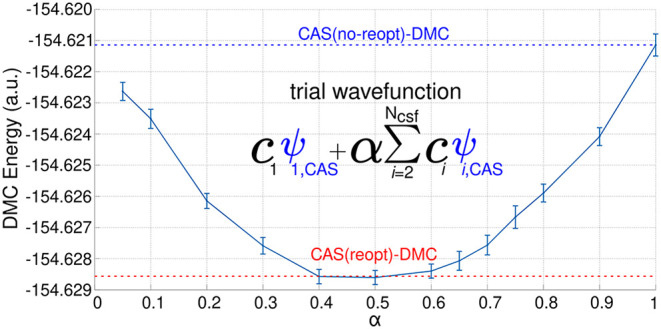

## I Introduction

Accurate electronic structure calculations are essential for providing benchmarks for assessing the performance of more approximate electronic structure methods, for designing force fields, and for machine learning applications.^1–6^ The diffusion Monte Carlo (DMC)^7–10^ and coupled cluster^11–14^ methods are two of the most widely used methods for benchmark calculations. While coupled cluster methods are systematically improvable through inclusion of higher-order excitation operators^15,16^ such calculations have steep computational scaling, and achieving well converged results requires the use of large basis sets, often combined with extrapolation to the complete basis set (CBS) limit. Localized orbital approximations such as the domain-based local pair natural orbital coupled-cluster [DLPNO-CCSD­(T)] ^17,18^ and pair-natural orbital local coupled cluster [PNO-LCCSD­(T)] ^19,20^ methods significantly reduce this cost. However, single-reference coupled cluster methods can fail to achieve chemical accuracy in systems with significant multireference character. The DMC method displays relatively low (O­(*N* ^3^)–O­(*N* ^4^)) scaling where *N* is the number of electrons, relatively weak dependence on the basis set, and a high degree of parallelization.^21–23^ DMC calculations are usually carried out using the fixed-node approximation,^24–26^ in which the nodal surface associated with electron exchange is imposed by use of a trial wave function. Most DMC studies use a single Slater determinant (SD) of Hartree–Fock or density functional theory (DFT) orbitals to impose the nodal surface. The resulting errors in energy differences, e.g., bond dissociation energies, due to the use of SD trial wave functions to describe the nodal surfaces can be sizable (i.e., several kcal/mol).^10,27,28^ One of the most used approaches to reduce the nodal surface error is to employ multideterminant (MD) trial wave functions.^10,27,29–33^ However, it is important to note that expanding the number of determinants in the trial wave function does not guarantee a decrease in the DMC energy.^31–34^

In a recent study^31^ of quinoidal molecules and related aromatic systems, we showed that using π-space CAS trial wave functions consistently improved energy differences from DMC calculations provided that the configuration interaction (CI) coefficients were reoptimized along with the parameters of a Jastrow factor^34–37^ using the variational Monte Carlo (VMC) procedure. Moreover, most coefficients smaller than ∼0.2 in magnitude were significantly reduced upon this reoptimization. This result is consistent with the configurations in the original CAS wave function playing an important role in describing both dynamic and static correlation effects, whereas upon reoptimization of the CI coefficients together with the parameters in the Jastrow factor, the Jastrow factor accounts for most of the dynamic correlation leaving the MD expansion to primarily describe the static correlation.

In this work, we extend this analysis to a broader range of unsaturated compounds. The systems examined are hydrocarbons and substituted hydrocarbons with one to four double bonds. In addition, we consider cyclobutadiene in both square and rectangular structures. The CAS-DMC calculations are carried out using both the original CASSCF wave functions as well as with CASSCF wave functions with the coefficients reoptimized in the VMC step, hereafter referred to as CAS­(no-reopt) and CAS­(reopt), respectively. The goal is to establish the relationship between the energy lowering in going from SD-DMC to CAS-DMC and the degree of multiconfigurational character in the system of interest.

## II Methodology

With the exception of cyclobutadiene, the geometries of all molecules studied in this work were optimized with the M*ø*ller-Plesset perturbation theory (MP2)^38^ method using the aug-cc-pVDZ basis set,^39,40^ with the optimizations being carried out using the Gaussian 16 program^41^ and employing the frozen-core approximation. The geometry of square cyclobutadiene was taken from ref 42. The CC bond lengths of that structure are referred to as *R*_sq_. The rectangular cyclobutadiene structures were generated by increasing the CC distance of two parallel CC bonds by a value δ and decreasing the CC distance of the other two CC bonds by δ, while keeping the CH bond lengths and CCH angles fixed. The values of δ considered range from 0.0 to 0.2 Å.

The DMC calculations were carried out with trial wave functions consisting of one or more Slater determinants multiplied by a Jastrow factor including 1-, 2-, and 3-body terms, with the parameters being optimized using VMC. B-spline basis functions were employed, with 10 parameters per spin channel for the 1- and 2-body Jastrow terms, and 26 parameters for the 3-body term. The Jastrow cutoff distances were fixed to 10 Bohr for the 1- and 2-body terms and 5 Bohr for the 3-body term. The Jastrow factor is symmetric with respect to electron exchange and thus does not impact the DMC energy. Three different choices—Hartree-Fock, π-space CASSCF, and π-space CASSCF with the coefficients reoptimized in the VMC step—were considered for the Slater determinant part of the trial wave function. The trial wave functions were determined using the cc-pVTZ or cc-pVQZ basis sets.^39^ The CASSCF calculations employed active spaces including all valence orbitals with a node in the molecular plane and were carried out using GAMESS.^43^ The DMC calculations were carried out at a time step of 0.001 au, with the total number of blocks being chosen to give statistical errors less than 0.25 kcal/mol when using 30 steps per block and 32,000 walkers. The time step of 0.001 au was chosen to ensure that the time-step bias is negligible compared to the targeted statistical uncertainty. The VMC and DMC calculations were carried out using QMCPACK.^44,45^

## III Results and Discussion

### III.A Benchmark Unsaturated Organic Molecules

Table 1 reports the differences between the SD-DMC energies and the two sets of CAS-DMC energies along with the coefficients of the two leading configurations of the CASSCF wave functions. The reader is referred to the SI for the corresponding configurations as well as a more complete description of the wave function.

**Table 1:** CAS-DMC – SD-DMC Energy Differences (kcal/mol) and Dominant Coefficients of the CAS Wave Functions before and after Re-Optimization in the VMC Step^a^ ^,^ ^b^ ^,^ ^c^

	CAS(no-reopt)-DMC			CAS(reopt)-DMC		
molecules and active space	energy change	1st coefficient	2nd coefficient	energy change	1st coefficient	2nd coefficient
ethylene (2,2)	–0.73 ± 0.22	0.977	–0.212	–3.00 ± 0.23	0.992	–0.127
pyrrole (6,5)	0.21 ± 0.25	0.964	–0.145	–4.27 ± 0.27	0.986	–0.092
1,4-cyclohexadiene (4,4)	–0.19 ± 0.30	0.958	0.200	–5.01 ± 0.29	0.984	0.123
furan (6,5)	0.84 ± 0.29	0.960	–0.159	–5.06 ± 0.30	0.983	–0.105
thiophene (6,5)	0.90 ± 0.32	0.959	–0.169	–4.79 ± 0.32	0.982	–0.114
1,3-cyclohexadiene (4,4)	–0.67 ± 0.28	0.953	–0.192	–5.35 ± 0.32	0.982	–0.121
benzene (6,6)	1.33 ± 0.31	0.939	–0.149	–6.18 ± 0.27	0.979	–0.094
*p*-xylene^a^ (10,8)	1.17 ± 0.32	0.939	–0.149	–6.36 ± 0.34	0.979	–0.091
hydroquinone^a^ (8,8)	0.68 ± 0.33	0.940	–0.148	–7.00 ± 0.32	0.976	0.098
1,2-benzoquinone^a^ (8,8)	1.71 ± 0.35	0.903	0.153	–11.79 ± 0.32	0.959	–0.126
paraquinone^a^ (8,8)	2.16 ± 0.35	0.908	0.159	–11.36 ± 0.37	0.957	–0.123
*p*-xylylene^a^ (8,8)	–2.37 ± 0.34	0.899	–0.210	–12.21 ± 0.35	0.953	–0.179
1,3-benzoquinone^a^ (8,8)	–2.94 ± 0.35	0.807	–0.293	–14.69 ± 0.33	0.873	–0.293

a Results for 1,3-cyclohexadiene and 1,4-cyclohexadiene were obtained using the cc-pVQZ basis set, while results for the other species were obtained using the cc-pVTZ basis set.

b The active space used for each species is indicated in parentheses as (*n*_elec_, *n*_orb_).

c Results from ref 31.

For several of the systems considered, the energies increased in going from SD-DMC to CAS­(no-reopt)-DMC. For all of these species, the magnitude of the second most important coefficient in the CASSCF wave function is less than 0.16. Significantly, the CAS­(reopt)-DMC calculations give energies lower than both the SD-DMC and CAS­(no-reopt)-DMC calculations for all systems considered. Reoptimizing the CI coefficients together with the parameters of the Jastrow results in an increase in magnitude of the coefficient of the dominant configurations for all systems considered. Moreover, it results in a decrease in magnitude of the coefficients of most of the minor configurations. In the case of 1,3-benzoquinone, which has significant diradical character, the coefficient of the second most important configuration has a value of −0.29 both before and after reoptimization. A least-squares fit of the data in Table 1 gives the following relation between the magnitude of the leading coefficients before and after reoptimization together with the Jastrow function

$$c_{i,\mathrm{re}‐\mathrm{opt}}=0.08x+2.59x^{2}-1.67x^{3}$$

where *x* represents the magnitude of the *c*_i_ coefficient directly from the CASSCF calculations. The coefficient data and this fit are shown in Figure 1. We do not attach physical significance to this functional form but include it as it is useful for predicting coefficient changes upon VMC optimization for unsaturated organic molecules other than those considered here.

**Figure 1:**
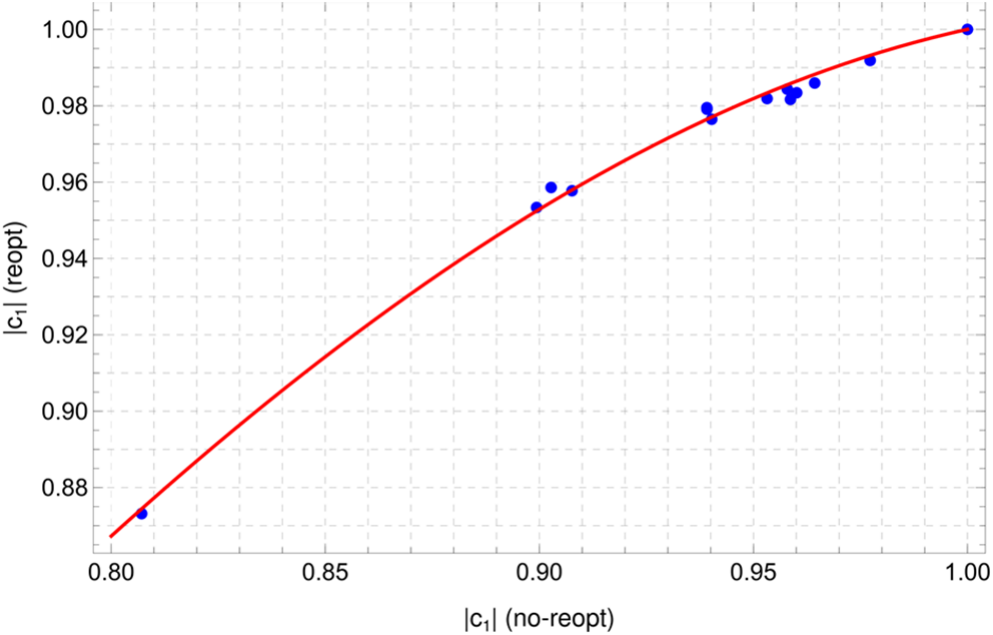
Value of the dominant coefficient in the CASSCF wave function after reoptimization in the VMC step vs the coefficient from the original CASSCF wave function for the molecules listed in Table 1. The data point at (1,1) is for a system with no double bonds.

CASSCF wave functions describe both dynamic and static correlation. When the CI coefficients and Jastrow factors are simultaneously optimized, the Slater determinant component of the trial wave function is more effective at describing the static correlation effects since the Jastrow recovers more of the dynamic correlation. It appears that in the absence of reoptimization of the CI coefficients together with the Jastrow factor, the CASSCF wave functions overestimate the distortion of the nodal surface from the Hartree–Fock nodal surface.

Figure 2 plots the SD-DMC–CAS­(reopt)-DMC energy difference vs the leading coefficient in both the CAS­(no-reopt) and CAS­(reopt) wave functions. A strong correlation is observed between the energy lowering and the magnitude of the leading coefficient in the CASSCF wave function. The energy lowering ranges from 3.0 kcal/mol for ethylene with a leading CAS coefficient of 0.977 to 17.7 kcal/mol for 1,3-benzoquinone with a leading coefficient of 0.807. Figure 2 includes a point for a leading coefficient of 1.0 and zero energy lowering. This is taken to correspond to a saturated molecule. In the present approach, using trial wave functions accounting for configuration mixing only in the π space, the trial wave function for a saturated molecule reduces to a Slater determinant of HF orbitals times the Jastrow factor. Finally, both sets of data in Figure 1 can be reasonably well fit by two straight lines. In the case of the original CASSCF coefficients, the crossover between the two regimes occurs for a leading coefficient of ∼0.91. The origin of this two-regime behavior in the energy lowering will be discussed later in the manuscript.

**Figure 2:**
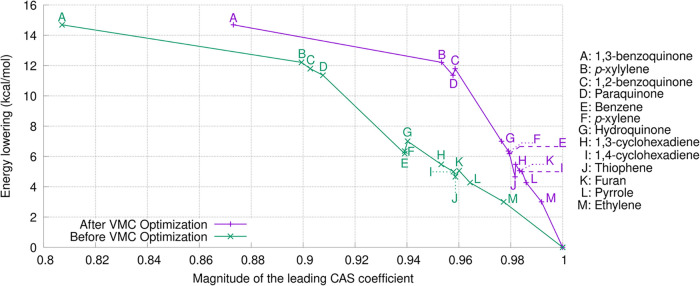
Energy lowering in going from SD-DMC to CAS­(reopt)-DMC vs the magnitude of the coefficient of the most important determinant in the wave function. Results are reported for the original coefficients from the CASSCF calculations (green data points) as well after reoptimization (purple data points) in the VMC step.

Previous studies have shown that in general MD-DMC calculations on saturated systems using large MD wave functions give significantly lower energies than SD-DMC calculations.^29,33,46,47^ Moreover, expanding the trial wave functions for unsaturated systems to include excitations from the σ orbitals and into the σ* orbitals is expected to further lower DMC energies for these species relative to those from the π-space CAS­(reopt)-DMC approach. In light of this, it is important to address the effectiveness of DMC calculations employing π-space CASSCF trial wave functions for addressing relevant chemical problems. In this context, we note that in ref 31 it was demonstrated that π-space CAS-DMC calculations provide substantially more accurate reaction energies for processes such as paraquinone + H_2_ → hydroquinone than obtained with SD-DMC. As another example, we consider the benzene + 3H_2_ → cyclohexane reaction. Ref 33 reported that the SD-DMC reaction energy for this process is in error by approximately 5 kcal/mol. If we combine the SD-DMC energies of cyclohexane, H_2_, and benzene from ref 33 with the present result for the energy lowering of benzene in going from SD-DMC to CAS­(reopt)-DMC, the error in this reaction energy is reduced to about 1 kcal/mol.

We note also that the calculations of ref 33 showed that the energy of benzene was lowered by about 16 kcal/mol when going from SD-DMC (using HF orbitals) to MD-DMC using a large selected CI trial wave function with on the order of 1 million Slater determinants. This trial wave function included excitations from all the occupied valence orbitals into the full virtual space. In contrast, our π-space CAS­(reopt)-DMC calculations gave an energy lowering of benzene of ∼6 kcal/mol compared to the SD-DMC result. Thus, although a sizable fixed-node error remains in the π-space CAS-DMC energy of benzene, this error is comparable to that in the SD­(HF)-DMC energy of cyclohexane. These results demonstrate the value of π-space CAS-DMC calculations for calculating chemically important energy differences.

### III.B Simple Scaling of the CI Coefficients Illustrated for Benzene

We next carried out a series of DMC calculations on benzene using trial wave functions in which all of the CASSCF coefficients, except the leading coefficient, were reduced by a factor α. The resulting energies are plotted in Figure 3, from which it is seen that the minimum energy is obtained for a scale factor of approximately 0.5. Strikingly, DMC calculations using this wave function as the trial wave function give an energy in excellent agreement with that obtained using the CAS­(reopt) trial wave function. If the individual coefficients of the configurations in the wave function were varied separately in the DMC calculations, an even lower DMC energy would result.

**Figure 3:**
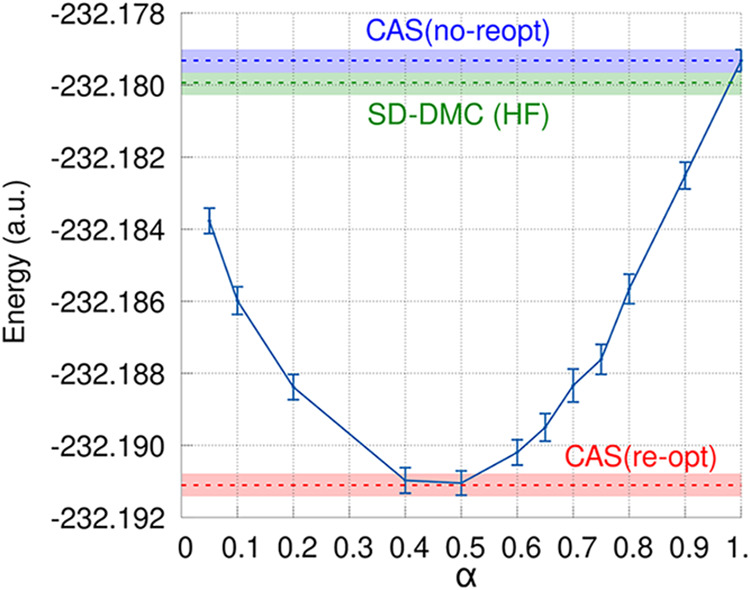
π-space CAS-DMC energy of benzene as a function of the scaling parameter α applied to the secondary coefficients of the trial wave function. The green, blue and red horizontal dashed lines denote the energies from the SD­(HF)-DMC, CAS­(no-opt)-DMC and CAS­(reopt)-DMC calculations, respectively. The statistical uncertainties in these energies are indicated by shading.

In the case of benzene we also carried out CI calculations allowing excitations from the entire occupied valence space into the entire virtual space. These calculations indicate that the ratios of the secondary configurations in the (π,π*) valence space to the dominant configuration are similar in the CAS­(reopt) and CI calculations. These results and details of the CI calculations are reported in the SI. Further studies are required to establish the generality of this intriguing result.

### III.C Tuning the Degree of Diradical Character with Geometric Distortion as Illustrated by Cyclobutadiene

We now consider the performance of π-Space CAS-DMC calculations for cyclobutadiene as it is distorted from the square to a rectangular structure. It is well-known that cyclobutadiene in its square structure is a diradical, for which the smallest “acceptable” wave function consists of two Slater determinants with coefficients equal in magnitude.^42,48–51^ As one pair of parallel C–C bonds is lengthened by an amount δ and the other pair shortened by δ, the degeneracy is lifted, with the two leading coefficients becoming 0.96 and −0.14, in a CAS­(4,4) calculation for δ = 0.2 Å. These coefficients are comparable to the two leading coefficients from the π-space CASSCF calculation on pyrrole (see Table 1).

Figure 4 reports the energy of cyclobutadiene as a function of δ. For each method considered, the energy at δ = 0.2 Å is taken as the reference. The SD-DMC and CCSD-F12 ^52,53^ potential energy curves are close to one another and increasingly deviate from the CASSCF and CAS-DMC curves as δ approaches zero. The CAS-DMC calculations give an energy difference between the potential energy minimum and the square structure in good agreement with the results of the CAS­(4,4)-multireference MP2 calculations of ref 42. For the square structure, the energy lowering when going from SD-DMC to π-space CAS-DMC is about 22 kcal/mol. This is greater than the energy lowering for any of the molecules considered in Table 1, consistent with the equal weights of the two dominant configurations for square butadiene. Interestingly, the CAS-DMC curves with and without coefficient reoptimization are very close. In other words, the energy lowering upon coefficient reoptimization is nearly constant (∼5 kcal/mol) independent of the value of δ. As δ is reduced from 0.2 to 0.125, the leading coefficient from the CAS calculations decreases from 0.962 to 0.944. While that may seem like a small change in the leading coefficient, the associated energy differences, as evaluated using the SD-DMC and CCSD­(T)-F12 methods, differ by about 2.5 kcal/mol from the CAS­(reopt)-DMC result.

**Figure 4:**
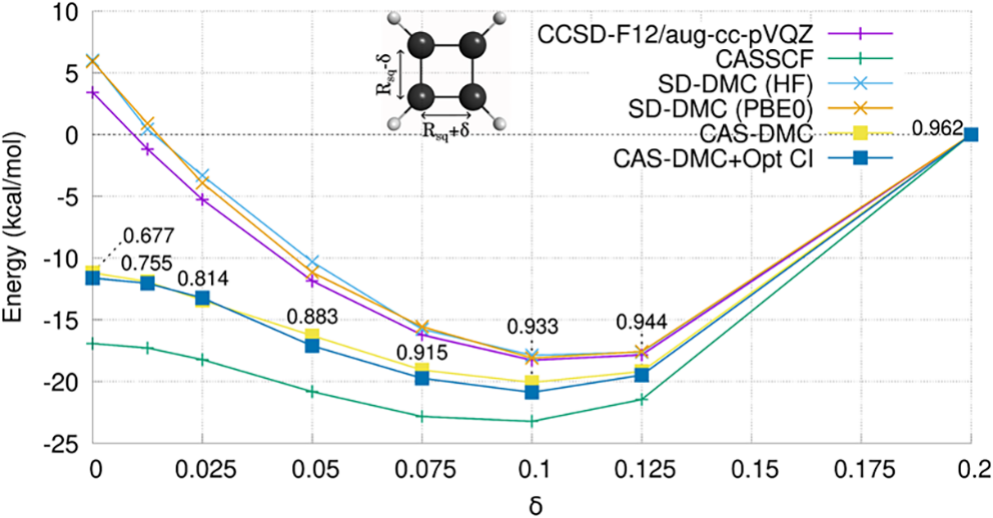
Energy of cyclobutadiene as a function of δ, which describes the distortion from the square to rectangular structure. For each method considered, the δ = 0.2 Å result is taken as the reference. The CAS coefficients before optimization are shown above the corresponding data points.

In Figure 5, we plot the energy lowering in going from SD­(HF)-DMC to π-space CAS­(reopt)-DMC as a function of the leading coefficient of the π-space CASSCF wave function. The energy lowering ranges from 4.2 kcal/mol for a leading coefficient of 0.96 (corresponding to δ = 0.2 Å) to 22.2 kcal/mol for a leading coefficient of 0.67 (corresponding to the square structure with δ = 0 Å). Comparing Figures 2 and 5 we see that there is fairly good agreement between the two sets of results over the range in which the coefficients overlap (i.e., for leading coefficients greater than 0.88). The main discrepancies between the results of the two figures are for paraquinone, 1,2-benzoquinone, and *p*-xylylene. These systems have leading CASSCF coefficient around 0.9 but experience about a 2 kcal/mol greater energy lowering (∼12 kcal/mol vs ∼10 kcal/mol) than does cyclobutadiene for a δ value giving a CASSCF coefficient of ∼0.9. A possible clue to the origin of this behavior is provided by examination of the leading CAS coefficients after reoptimization together with the parameters in the Jastrow factors. The change in the leading coefficient upon reoptimization is greater for the above three mentioned molecules than for cyclobutadiene distorted to a structure (δ ≈ 0.0625) with a leading coefficient of 0.90.

**Figure 5:**
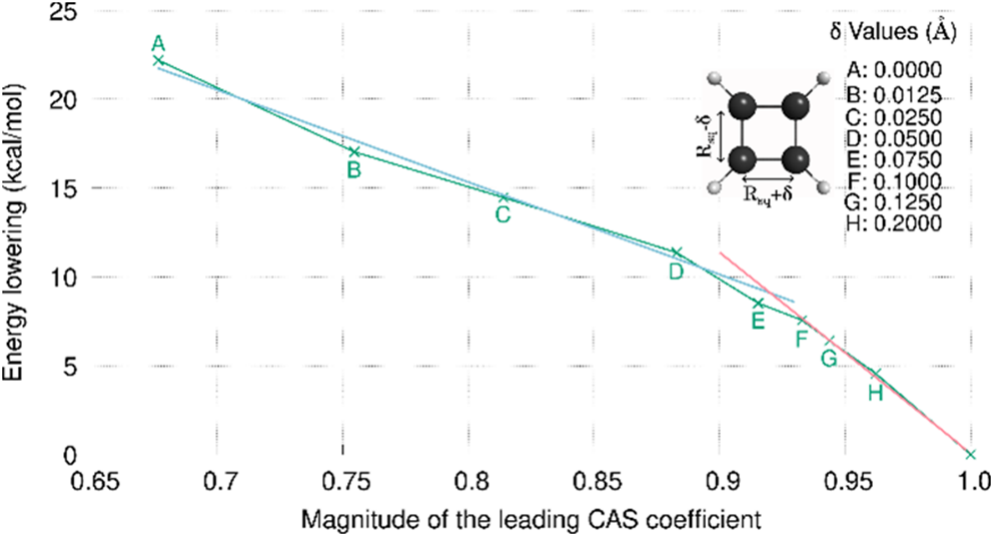
Energy lowering from SD­(HF)-DMC to π-space CAS­(reopt)-DMC for cyclobutadiene as a function of the leading coefficient of the π-space CASSCF wave function. This coefficient, in turn depends on δ as shown in Figure S2. Straight lines have been added to highlight the two limiting regimes discussed in the text. The data point (1,0) is imposed rather than being from an actual calculation.

Figure 5 also shows that for cyclobutadiene the SD-DMC–CAS­(reopt)-DMC energy lowering vs the leading CI coefficient displays two limiting regimes, one for coefficients greater than ∼0.91 and another for coefficients less than ∼0.91. In both regimes, the data are well represented by linear curves, with different slopes. Similar behavior was seen for the group of molecules considered earlier in the paper and reported in Figure 1, although there is more scatter in the data points due to the use of different molecules. This two-regime behavior was previously noted in ref 32. for H_4_ when the molecule was distorted from a square to a rectangular structure. There it was rationalized in terms of a simple 2 × 2 configuration interaction (CI) model involving the two leading configurations which contribute equally in the square structure. In this model, the key parameters are the integral *K* describing the coupling between the two configurations and the energy gap, 2Δ, between the two noninteracting configurations The gap is equal to twice the HOMO/LUMO orbital energy difference in the lowest energy triplet state as calculated from ROHF calculation using Guest-Saunders^54^ coupling. The two limiting regimes correspond to the Δ ≫ *K* and Δ ≪ *K* limits, with the latter representing the strongly correlated case. Figure 6 plots the value of the leading CI coefficient of cyclobutadiene from the CAS­(4,4) calculations vs the orbital energy gap as determined from an ROHF/3-21G^55^ calculations with Guest-Saunders coupling for the lowest energy triplet state. Both the leading CI coefficient and the orbital gap vary with the degree of distortion from the square structure. Notably, the crossover between the two regimes in Figure 6 occurs at approximately the same value of the CASSCF coefficient (∼0.91) as found in Figure 5. In the SI, we show the corresponding figure using coefficients from CAS­(2,2) calculations, showing that the analytical expression derived from the 2 × 2 CI reported in ref 32. quantitatively accounts for the dependence of the magnitude of the leading CI coefficient on the orbital energy gap.

**Figure 6:**
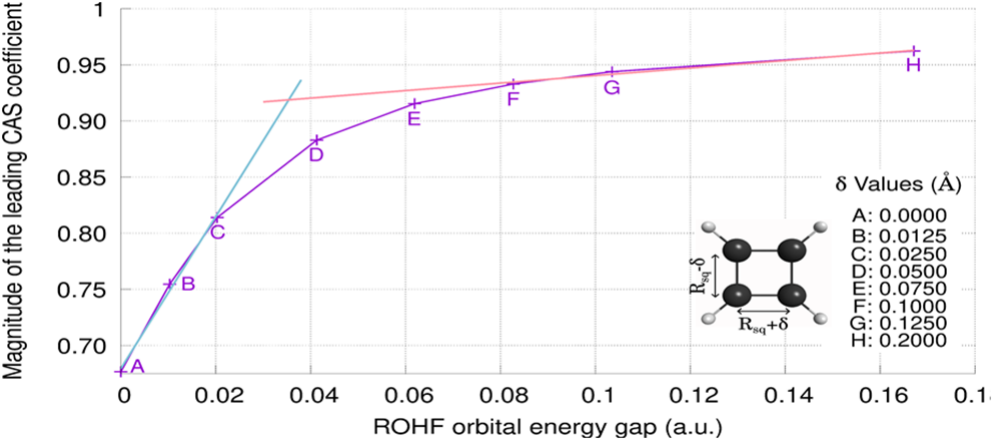
Coefficient of the leading configuration of π-space CAS­(4,4) on cyclobutadiene vs the orbital energy gap (a.u.) from ROHF/3–21G calculations of the lowest triplet state using Guest-Saunders coupling. Results obtained from calculations with δ ranging from 0.0 to 0.2.

We also explored simple rescaling of all secondary coefficients in the CAS­(4,4) wave function of square cyclobutadiene. Because the two leading coefficients contribute with equal weight, this involves scaling of all remaining coefficients. As seen from Figure 7, applying a scale factor of about 0.45 gives a MD-DMC energy slightly lower than that obtained from coefficient optimization at the VMC step. When one renormalizes the wave function, the magnitudes of the two (degenerate) leading configurations increase.

**Figure 7:**
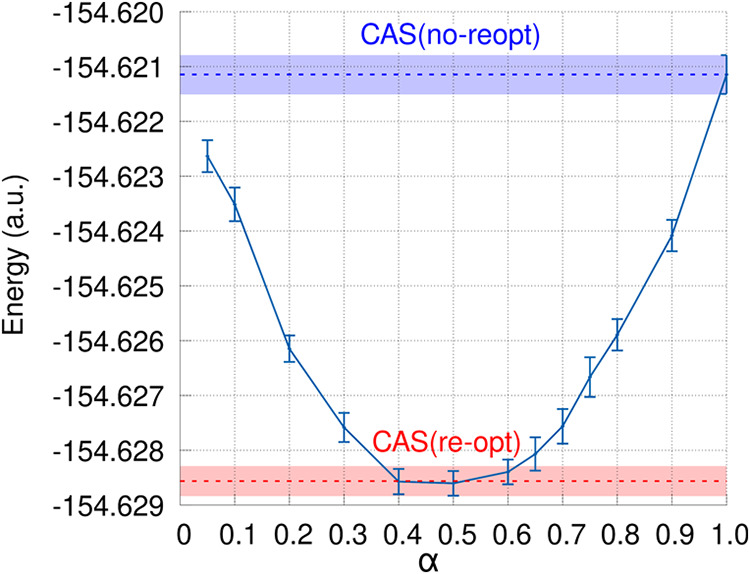
CAS­(4,4)-DMC energy of cyclobutadiene with the geometry given by δ = 0.0 Å as a function of the scaling factor applied to all coefficients except the two dominant ones. The blue and red horizontal lines indicate the energies of the CAS­(no-reopt)-DMC and CAS­(reopt)-DMC calculations, respectively. The shaded bands represent the statistical uncertainties.

As demonstrated above for benzene and cyclobutadiene one can obtain DMC energies similar to those obtained with CAS­(reopt) trial wave functions by a 50% reduction in the magnitude of the coefficients of the minor configuration in the CAS trial wave function. While investigating the generality of this finding by examining a large number of additional compounds is beyond the scope of the present study, we have examined this for pyrrole and paraquinone, for which we also find that a simple 0.5 scaling of the secondary coefficients in the CAS trial wave functions results in essentially the same DMC energies as using the CAS­(reopt) trial wave functions. These results are reported in the SI.

## IV Conclusion

In this work we examined for several unsaturated organic molecules the energy lowering in going from SD-DMC to MD-DMC, employing π-space CASSCF trial wave functions. We demonstrate that, provided the CI coefficients are reoptimized in the VMC step, π-space CAS-DMC calculations can give significantly improved energy differences compared to the SD-DMC results for both chemical reactions such as benzene + 3H_2_ → cyclohexane and for geometrical distortions as in square to rectangular cyclobutadiene. We find a strong correlation between the magnitude of the leading Slater-determinant coefficient in the π-space CASSCF wave function and the corresponding CAS-DMC–SD-DMC energy lowering provided that the coefficients in the wave function are reoptimized with the Jastrow parameters. This correlation is observed across various unsaturated systems and extends to structural distortions within a given molecule, as illustrated by the cyclobutadiene case. Moreover, it allows for the estimation of the π-space CAS­(reopt)-DMC energies of molecules similar to those considered in the present study without actually carrying out the calculations. In addition, it is shown that for ethylene, benzene, cyclobutadiene, pyrrole, and paraquinone similar energy lowering in CAS-DMC calculations is achieved through a simple rescaling of the coefficients of the minor configurations, offering a computationally efficient approximation. In recent years, there has been growing interest in gradient-based approaches for optimizing the nodal surface in DMC calculations.^56–59^

## Supplementary Material



## Notes

Ten most important CAS configurations for all systems considered; CAS configurations of cyclobutadiene before and after VMC optimization for each value of δ considered; DMC energies of cylobutadiene using different trial wave functions together with HF, CAS­(2,2), CAS­(4,4), and CCSD­(T)-F12 energies, and the ROHF/3-21G HOMO/LUMO gap of the lowest triplet state using the Guest and Saunders coupling; energy lowering of cyclobutadiene when going from SD­(HF)-DMC to π-space CAS-DMC before and after coefficient optimization in VMC as a function of the leading coefficient of the π-space CASSCF wave function energy lowering of cylobutadiene when going from π-space CAS-DMC to SD­(HF)-DMC as a function of the distortion δ; CAS­(2,2) CI coefficients vs orbital energy gap for cyclobutadiene compared to the 2 × 2 analytical model; and DMC energy of pyrrole and paraquinone obtained using π-space CAS-DMC trial wave functions (PDF)
