# Supplementary material for: Use of π‑Space CASSCF Trial Wave Functions in DMC Calculations of Unsaturated Organic Molecules
Source: J Phys Chem A. 2026 Jul 20;130(30):5833–9. doi: 10.1021/acs.jpca.6c02019 (PMC13430682; doi:10.1021/acs.jpca.6c02019)
Supplement: Supplementary file 1 [file jp6c02019_si_001.pdf]

**Supporting Information for Use of π-space CASSCF Trial Wave Functions in DMC Calculations of Unsaturated Organic Molecules**

N. Mauger* and K. D. Jordan*
Department of Chemistry, University of Pittsburgh
Pittsburgh, PA 15218, United States

Table S1. Leading configurations in the CASSCF wave function before and after VMC optimization for all studied molecules.

| 1,3-benzoquinone | | | p–xylylene | | |
|---|---|---|---|---|---|
| Configuration | Coefficient before optimization | Coefficient after optimization | Configuration | Coefficient before optimization | Coefficient after optimization |
| 22220000 | 0.807099 | 0.873136 | 22220000 | 0.899362 | 0.953338 |
| 22202000 | -0.293420 | -0.293638 | 22202000 | -0.210044 | -0.179152 |
| 22112000 | 0.188825 | 0.164166 | 21211010 | -0.163679 | -0.107594 |
| 21221000 | -0.202491 | -0.153591 | 22111100 | 0.132623 | 0.091855 |
| 22201010 | 0.176238 | 0.153047 | 22020200 | -0.113835 | -0.076656 |
| 12212000 | 0.110007 | 0.080805 | 12121100 | 0.086539 | 0.056349 |
| 21211100 | -0.099246 | -0.074044 | 12211001 | -0.082024 | -0.045885 |
| 22111010 | -0.095448 | -0.071136 | 12120101 | 0.080084 | 0.044664 |
| 21211001 | 0.103526 | 0.067434 | 22110101 | 0.068523 | 0.043028 |
| 22022000 | -0.069522 | -0.062287 | 20220020 | -0.075774 | -0.042380 |

| 1,2-benzoquinone | | | Paraquinone | | |
|---|---|---|---|---|---|
| Configuration | Coefficient before optimization | Coefficient after optimization | Configuration | Coefficient before optimization | Coefficient after optimization |
| 22220000 | 0.902753 | 0.958577 | 22220000 | 0.907633 | 0.957728 |
| 22202000 | -0.147210 | -0.125705 | 22022000 | -0.147013 | -0.123218 |
| 22111100 | 0.152996 | 0.108350 | 22111100 | 0.158969 | 0.120727 |
| 21211010 | -0.101424 | -0.071792 | 21121010 | 0.127374 | 0.083554 |
| 22022000 | -0.077020 | -0.061203 | 22200200 | -0.111155 | -0.082686 |
| 22201010 | -0.079480 | -0.057501 | 11221010 | -0.106149 | -0.064213 |
| 20222000 | -0.073457 | -0.052276 | 22202000 | -0.069467 | -0.054822 |
| 11221100 | -0.081923 | -0.051142 | 12121001 | -0.086359 | -0.05359 |
| 20221010 | 0.079566 | 0.049181 | 12211100 | 0.070818 | 0.052096 |
| 12121010 | 0.069039 | 0.043321 | 22110101 | 0.076973 | 0.049394 |

| Benzene | | | p-xylene | | |
|---|---|---|---|---|---|
| Configuration | Coefficient before optimization | Coefficient after optimization | Configuration | Coefficient before optimization | Coefficient after optimization |
| 222000 | 0.939127 | 0.979487 | 22222000 | 0.939146 | 0.979086 |
| 202200 | -0.149307 | -0.090734 | 22202200 | -0.148732 | -0.091455 |
| 220020 | -0.149307 | -0.090734 | 22220020 | -0.145034 | -0.088187 |
| 211110 | -0.138318 | -0.088467 | 22211110 | 0.135955 | 0.087540 |
| 112101 | -0.110876 | -0.060043 | 22211110 | -0.113532 | -0.058197 |
| 121011 | 0.110876 | 0.060043 | 22112101 | 0.105260 | 0.057881 |
| 211110 | 0.115325 | 0.057094 | 22121011 | 0.097205 | 0.054630 |
| 202020 | -0.033982 | -0.033353 | 22220200 | -0.036535 | -0.036774 |
| 220200 | -0.033982 | -0.033353 | 22202020 | -0.030193 | -0.029577 |
| 022020 | -0.042054 | -0.027497 | 12221011 | 0.047500 | 0.024579 |

| Hydroquinone | | | 1,3-cyclohexadiene (cc-pVQZ) | | |
|---|---|---|---|---|---|
| Configuration | Coefficient before optimization | Coefficient after optimization | Configuration | Coefficient before optimization | Coefficient after optimization |
| 22222000 | 0.940262 | 0.976456 | 2200 | 0.953195 | 0.981877 |
| 22211110 | 0.132967 | 0.09806 | 2020 | -0.173846 | -0.120772 |
| 22202200 | -0.148126 | -0.097575 | 1111 | -0.191554 | -0.111946 |
| 22220020 | -0.136304 | -0.087524 | 0220 | -0.089371 | -0.056041 |
| 22211110 | -0.112239 | -0.060434 | 2002 | -0.078186 | -0.046354 |
| 22112101 | -0.099283 | -0.058968 | 0202 | -0.077583 | -0.042828 |
| 22121011 | -0.086069 | -0.049251 | 1111 | -0.047601 | -0.032282 |
| 22220200 | -0.045442 | -0.043263 | 0022 | 0.040992 | 0.017517 |
| 12221011 | -0.058546 | -0.029794 | 1210 | 0.013772 | -0.014879 |
| 22022200 | -0.036329 | -0.025676 | 2101 | -0.006562 | 0.008916 |

| 1,4-cyclohexadiene (cc-pVQZ) | | | Thiophene | | |
|---|---|---|---|---|---|
| Configuration | Coefficient before optimization | Coefficient after optimization | Configuration | Coefficient before optimization | Coefficient after optimization |
| 2200 | 0.957921 | 0.984340 | 22200 | 0.958654 | 0.981662 |
| 1111 | 0.200064 | 0.123807 | 22020 | -0.168788 | -0.114431 |
| 2020 | -0.113487 | -0.070603 | 12111 | -0.123092 | -0.070772 |
| 0220 | -0.096762 | -0.059376 | 21111 | -0.085643 | -0.064196 |
| 2002 | -0.094135 | -0.058977 | 21111 | -0.096594 | -0.053341 |
| 0202 | -0.096475 | -0.056897 | 20220 | -0.053118 | -0.049226 |
| 0022 | 0.042119 | 0.016775 | 22002 | -0.065261 | -0.045967 |
| 1111 | -0.013274 | -0.015497 | 20202 | -0.061734 | -0.041630 |
| | | | 21210 | 0.013793 | -0.040732 |
| | | | 11202 | -0.063250 | -0.037538 |

| Furan | | | Pyrrole | | |
|---|---|---|---|---|---|
| Configuration | Coefficient before optimization | Coefficient after optimization | Configuration | Coefficient before optimization | Coefficient after optimization |
| 22200 | 0.960109 | 0.983394 | 22200 | 0.964318 | 0.985949 |
| 22020 | -0.159144 | -0.104975 | 22020 | -0.145147 | -0.092288 |
| 21111 | 0.138492 | 0.077529 | 21111 | 0.093781 | 0.059505 |
| 21111 | 0.080743 | 0.057089 | 21111 | 0.099097 | 0.053805 |
| 22002 | -0.074744 | -0.049538 | 20202 | -0.087138 | -0.052574 |
| 20202 | -0.081422 | -0.049392 | 12111 | 0.090003 | 0.050875 |
| 12111 | -0.078124 | -0.046063 | 22002 | -0.069172 | -0.048316 |
| 20220 | -0.063874 | -0.043646 | 20220 | -0.058140 | -0.044861 |
| 12210 | 0.005340 | 0.032980 | 02220 | -0.044316 | -0.027140 |
| 21210 | -0.013110 | 0.030704 | 11202 | -0.050414 | -0.026777 |

| Ethylene | | |
|---|---|---|
| Configuration | Coefficient before optimization | Coefficient after optimization |
| 20 | 0.977274 | 0.991864 |
| 02 | -0.211980 | -0.127302 |

Table S2. Cyclobutadiene CASSCF wave functions before and after VMC optimization.

| δ =0.000 | | | δ =0.0125 | | |
|---|---|---|---|---|---|
| Configuration | Coefficient before optimization | Coefficient after optimization | Configuration | Coefficient before optimization | Coefficient after optimization |
| 2200 | 0.676599 | 0.693825 | 2200 | 0.754608 | 0.788387 |
| 2020 | -0.676599 | -0.693825 | 2020 | -0.588253 | -0.584158 |
| 1111 | 0.234434 | 0.161760 | 1111 | 0.241021 | 0.165382 |
| 1111 | -0.135343 | -0.092540 | 1111 | -0.118646 | -0.082593 |
| 0022 | 0.074950 | 0.035250 | 0022 | 0.073507 | 0.032873 |
| 0202 | -0.074950 | -0.035250 | 0202 | -0.074886 | -0.035824 |
| | | | 0220 | -0.026939 | -0.020202 |
| | | | 2002 | -0.024889 | -0.016124 |

| δ =0.025 | | | δ =0.050 | | |
|---|---|---|---|---|---|
| Configuration | Coefficient before optimization | Coefficient after optimization | Configuration | Coefficient before optimization | Coefficient after optimization |
| 2200 | 0.813923 | 0.863771 | 2200 | 0.882927 | 0.923045 |
| 2020 | -0.503117 | -0.465111 | 2020 | -0.370975 | -0.338751 |
| 1111 | 0.242717 | 0.166272 | 1111 | 0.237066 | 0.151034 |
| 1111 | -0.102098 | -0.071460 | 1111 | -0.075934 | -0.055084 |
| 2002 | -0.046243 | -0.039858 | 2002 | -0.074614 | -0.055116 |
| 0220 | -0.049828 | -0.035656 | 0220 | -0.079153 | -0.050256 |
| 0202 | -0.073652 | -0.031902 | 0202 | -0.070016 | -0.033022 |
| 0022 | 0.070855 | 0.030680 | 0022 | 0.064155 | 0.027147 |

| δ =0.075 | | | δ =0.100 | | |
|---|---|---|---|---|---|
| Configuration | Coefficient before optimization | Coefficient after optimization | Configuration | Coefficient before optimization | Coefficient after optimization |
| 2200 | 0.915393 | 0.956739 | 2200 | 0.932883 | 0.970403 |
| 2020 | -0.287610 | -0.230824 | 2020 | -0.234391 | -0.175596 |
| 1111 | 0.227200 | 0.144162 | 1111 | 0.216903 | 0.130099 |
| 2002 | -0.088753 | -0.059761 | 2002 | -0.095174 | -0.065025 |
| 1111 | -0.059382 | -0.040452 | 0220 | -0.097235 | -0.061974 |
| 0220 | -0.092357 | -0.061913 | 0202 | -0.064357 | -0.031956 |
| 0202 | -0.066797 | -0.032858 | 1111 | -0.048944 | -0.030670 |
| 0022 | 0.057634 | 0.021632 | 0022 | 0.051899 | 0.022988 |

| δ =0.125 | | | δ =0.200 | | |
|---|---|---|---|---|---|
| Configuration | Coefficient before optimization | Coefficient after optimization | Configuration | Coefficient before optimization | Coefficient after optimization |
| 2200 | 0.943854 | 0.976810 | 2200 | 0.962274 | 0.986532 |
| 2020 | -0.198194 | -0.142163 | 2020 | -0.136253 | -0.090769 |
| 1111 | -0.207058 | -0.124849 | 1111 | 0.180721 | 0.104221 |
| 0220 | -0.098054 | -0.064383 | 0220 | -0.091573 | -0.055014 |
| 2002 | -0.097526 | -0.063546 | 2002 | -0.094309 | -0.052888 |
| 0202 | -0.062466 | -0.030118 | 0202 | -0.058286 | -0.028997 |
| 1111 | -0.041941 | -0.027134 | 1111 | -0.029951 | -0.029098 |
| 0022 | 0.046898 | 0.014759 | 0022 | 0.035059 | 0.011897 |

Table S3. DMC energies (a.u.) of cyclobutadiene using different trial wave functions as a function of δ.

| δ | Method | | | |
|---|---|---|---|---|
| | SD-DMC (PBE0)/cc-pVTZ | SD-DMC (HF)/cc-pVTZ | CAS-DMC/cc-pVTZ | CAS-DMC (reopt)/cc-pVTZ |
| 0.000 | -154.5977 ± 0.0003 | -154.5932 ± 0.0004 | -154.6211± 0.0004 | -154.6286 ± 0.0003 |
| 0.0125 | -154.6057 ± 0.0003 | -154.6021 ± 0.0004 | -154.6223 ± 0.0003 | -154.6293 ± 0.0003 |
| 0.025 | -154.6133 ± 0.0003 | -154.6081 ± 0.0003 | -154.6247 ± 0.0003 | -154.6311 ± 0.0003 |
| 0.050 | -154.6249 ± 0.0003 | -154.6192 ± 0.0003 | -154.6293 ± 0.0003 | -154.6373 ± 0.0003 |
| 0.075 | -154.6319 ± 0.0003 | -154.6279 ± 0.0003 | -154.6337 ± 0.0003 | -154.6415 ± 0.0003 |
| 0.100 | -154.6360 ± 0.0004 | -154.6312 ± 0.0003 | -154.6353 ± 0.0003 | -154.6433 ± 0.0003 |
| 0.125 | -154.6351 ± 0.0003 | -154.6309 ± 0.0003 | -154.6339 ± 0.0003 | -154.6411 ± 0.0003 |
| 0.200 | -154.6071 ± 0.0003 | -154.6028 ± 0.0003 | -154.6033 ± 0.0003 | -154.6100 ± 0.0003 |

Table S4. HF and CASSCF energies (a.u.) of cyclobutadiene as a function of δ. The ROHF energies were calculated using the Guest and Saunders coupling.

| δ | Method | | | | |
|---|---|---|---|---|---|
| | ROHF – HOMO/LUMO gap from lowest triplet/3-21G | HF/cc-pVTZ | CASSCF(2,2)/cc-pVTZ | CASSCF (4,4)/cc-pVTZ | CCSD-F12/aVQZ |
| 0.000 | 0.0 | -153.6447 | -153.6851 | -153.7494 | -154.3775 |
| 0.0125 | 0.0103 | -153.6542 | -153.6860 | -153.7499 | -154.3848 |
| 0.025 | 0.0206 | -153.6627 | -153.6883 | -153.7514 | -154.3913 |
| 0.050 | 0.0413 | -153.6766 | -153.6948 | -153.7556 | -154.4018 |
| 0.075 | 0.0619 | -153.6865 | -153.7006 | -153.7588 | -154.4088 |
| 0.100 | 0.0827 | -153.6923 | -153.7038 | -153.7594 | -154.4120 |
| 0.125 | 0.1035 | -153.6937 | -153.7036 | -153.7566 | -154.4114 |
| 0.200 | 0.1671 | -153.6695 | -153.6765 | -153.7224 | -154.3829 |

In order to explore whether our findings concerning the relative magnitudes of the CI coefficients from valence (π,π*) CAS(reopt) calculations potentially carry over to CI calculations, we also carried out for benzene selected CI (sCI) calculations employing the ccECP effective core potential and associated double-zeta basis set.[1] These calculations were carried out using QuantumPackage [2] and allowed excitations from all valence orbitals into the entire virtual space with several iterations of natural orbital generation. These calculations gave coefficients of the most important $\pi \rightarrow \pi^*$ excitations that are considerably smaller than those found in the CAS(reopt) calculations. However, the ratios of the most important secondary configurations in the π/π* valence space to the dominant configurations from the sCI calculations are in fairly good agreement with the corresponding ratios from the CAS(reopt) calculations. It should be noted that much of the dynamic correlation associated with excitation of σ electrons in the sCI calculations is recovered through the Jastrow factor employed in the CAS(reopt) calculations. We speculate that correlation effects involving the σ electrons provide a dielectric screening of the π electrons, reducing the importance of the secondary configurations in the (π,π*) valence space in both the CAS(reopt) and sCI calculations.

Table S5. Absolute values of the ratios of the coefficients of the leading secondary configurations in the wave function to the coefficient of the dominant configurations in selected CI (sCI) and CAS(reopt) trial wavefunctions for benzene.

| Configuration | sCI | CAS(reopt) |
|---|---|---|
| 202200 | 0.092 | 0.093 |
| 220020 | 0.092 | 0.093 |
| 211110 | 0.069 | 0.090 |
| 112101 | 0.047 | 0.061 |
| 121011 | 0.047 | 0.061 |

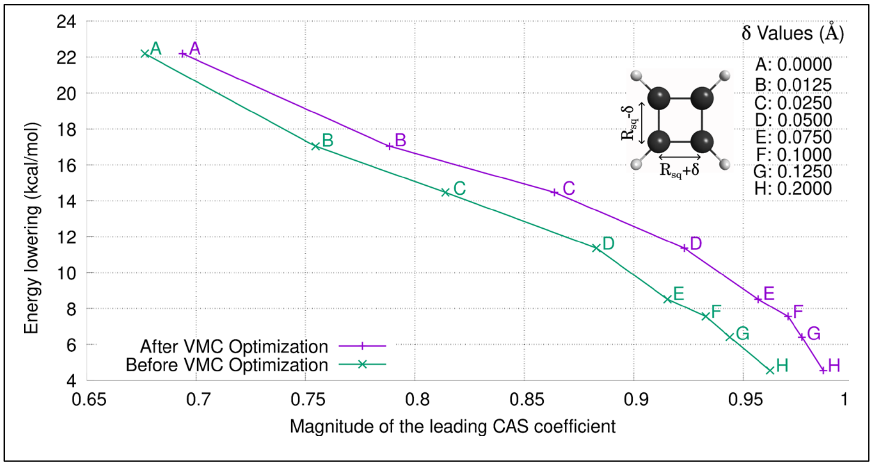


Fig. S1. Energy lowering in going from SD(HF)-DMC to π-space CAS-DMC as a function of the leading coefficient of the π-space CASSCF wave function before and after VMC optimization.

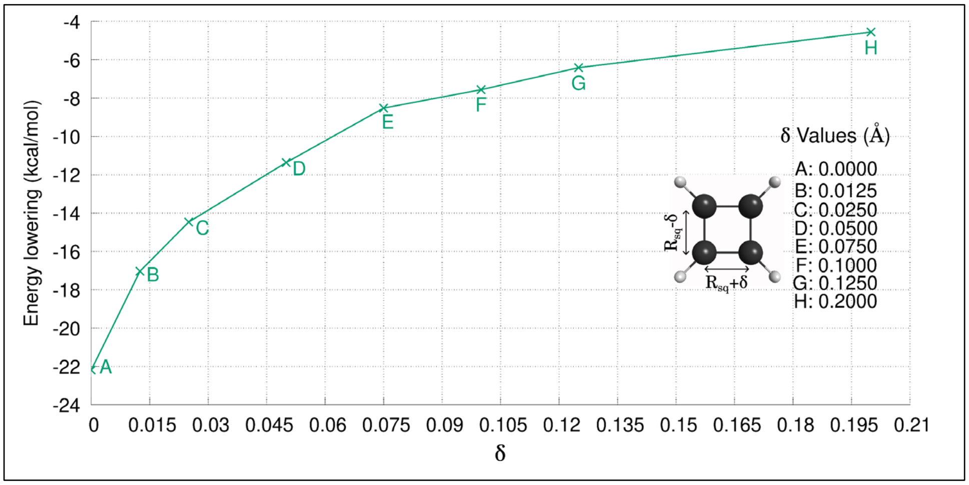


Fig. S2. Energy lowering in going from SD(HF)-DMC to π-space CAS(reopt)-DMC as a function of δ.

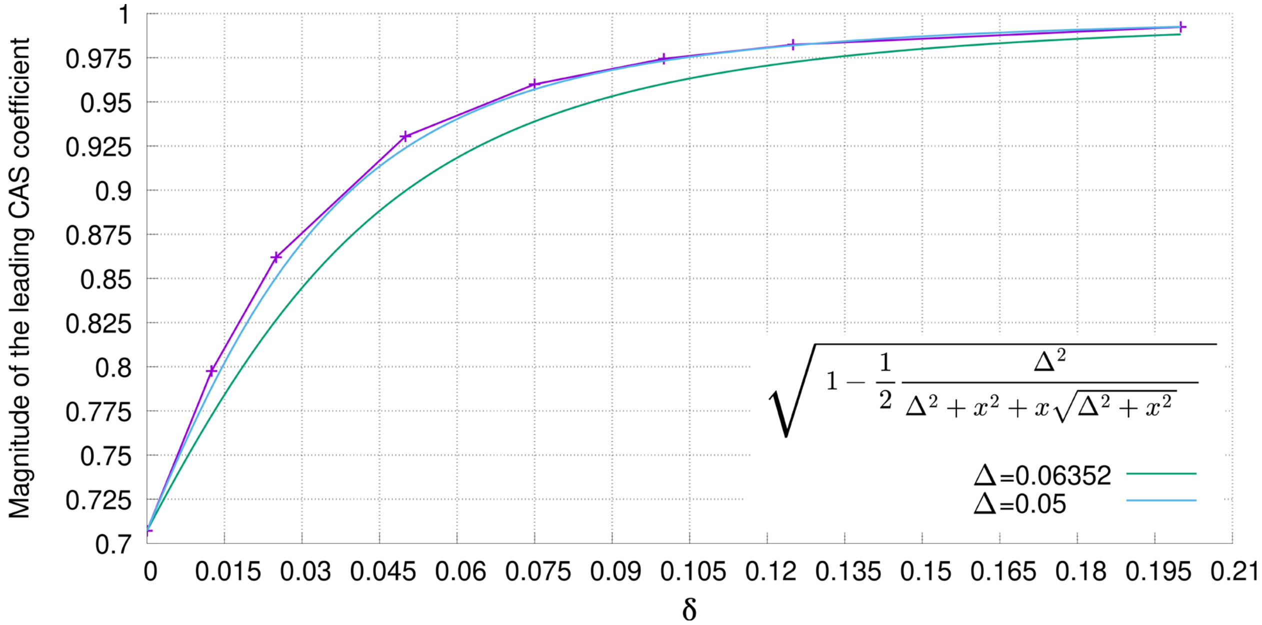


Fig. S3. Magnitude of the dominant CASSCF coefficient of cyclobutadiene obtained from CAS(2,2) calculations as a function of δ. The analytical expression from the 2×2 CI model is shown for comparison, demonstrating quantitative agreement across the entire range with Δ=0.05 a.u. (blue), whereas the value extracted from the cc-pVTZ calculations is Δ=0.06352 a.u. (green), where 2Δ is the energy difference between the two configurations.

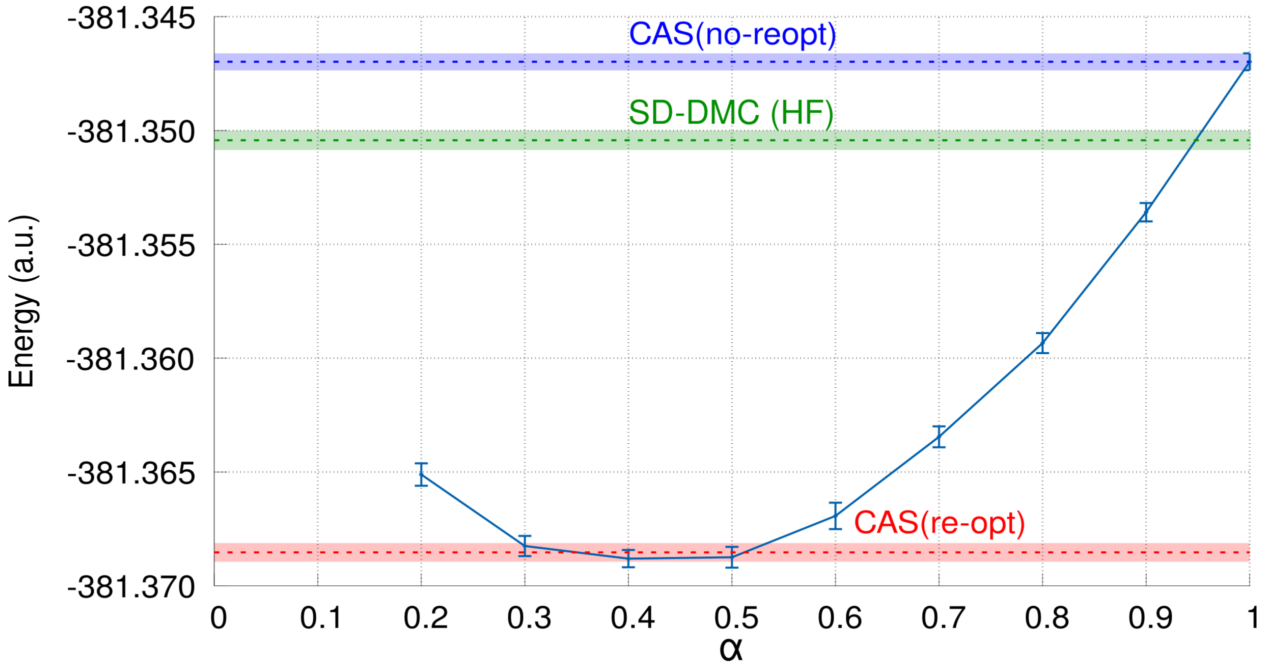


Fig. S4. CAS(8,8)-DMC energy of paraquinone as a function of the scaling factor α applied to the secondary coefficients of the trial wave function. The green, blue, and red horizontal dashed lines denote the energies from the SD(HF)-DMC, CAS(no-opt)-DMC and CAS(reopt)-DMC calculations, respectively. The statistical uncertainties in these energies are indicated by shading.

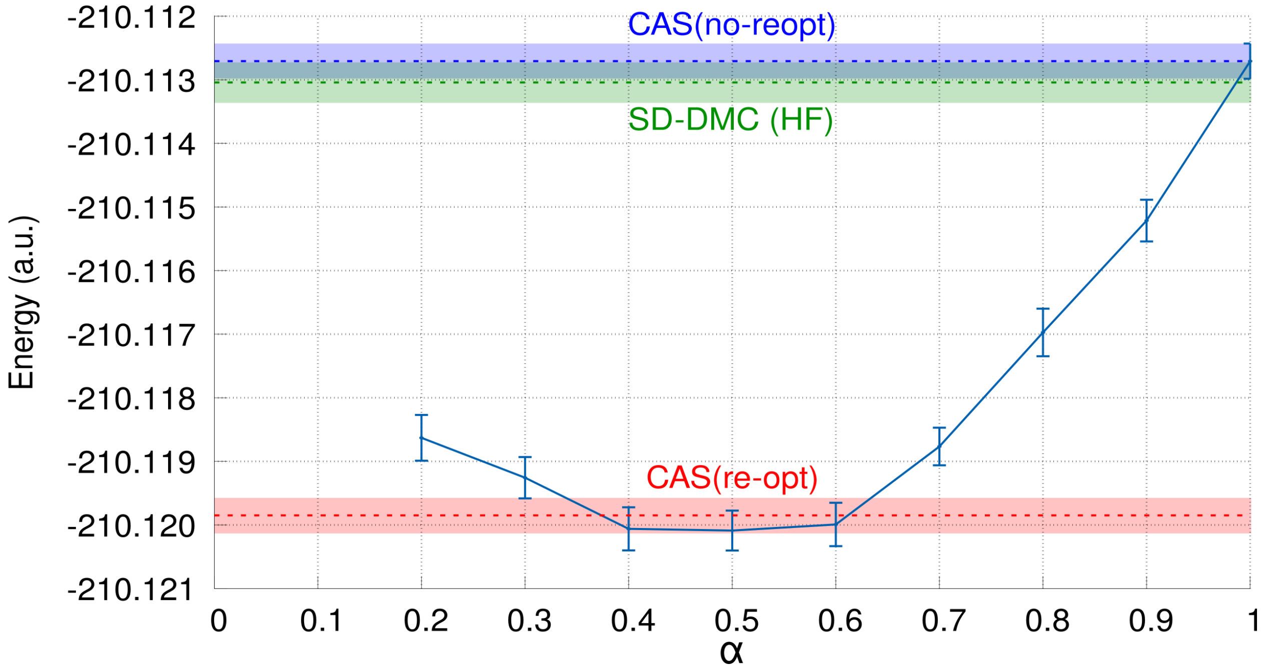


Fig. S5. CAS(6,5)-DMC energy of pyrrole as a function of the scaling factor α applied to the secondary coefficients of the trial wave function. The green, blue and red horizontal dashed lines denote the energies from the SD(HF)-DMC, CAS(no-opt)-DMC and CAS(reopt)-DMC calculations, respectively. The statistical uncertainties in these energies are indicated by shading.

**References**

1. Bennett, M. C.; Melton, C. A.; Annaberdiyev, A.; Wang, G.; Shulenburger, L.; Mitas, L.; A new generation of effective core potentials for correlated calculations. *J. Chem. Phys.*, *147,* 224106 (2017).

2. Garniron, Y.; Scemama, A.; Giner, E.; Caffarel, M.; Loos, P. F.; Selected configuration interaction dressed by perturbation. *J. Chem. Phys.*, *149,* 064103 (2018).
